# Supplementary material for: Resolving the full spectrum of human genome variation using Linked-Reads
Source: Genome Res. 2019 Apr;29(4):635–45. doi: 10.1101/gr.234443.118 (PMC6442396; doi:10.1101/gr.234443.118)
Supplement: Supplemental Material [file supp_gr.234443.118_Supplemental_longranger-2.2.zip › longranger-2.2.tar.bz2!/lr2.2-public/lib/stan/cmdstan/stan/doc/api/html/structstan_1_1gm_1_1generate__init__visgen.html]

Stan: stan::gm::generate\_init\_visgen Struct Reference


|  |  |
| --- | --- |
|  | Stan  2.5.0  probability, sampling & optimization |


- Main Page
- Related Pages
- Namespaces
- Classes
- Files

- Class List
- Class Index
- Class Hierarchy
- Class Members

All Classes Namespaces Files Functions Variables Typedefs Enumerations Enumerator Friends Macros Pages

- stan
- gm
- generate\_init\_visgen

Public Member Functions |
Public Attributes |
List of all members

stan::gm::generate\_init\_visgen Struct Reference

`#include <generator.hpp>`

Inheritance diagram for stan::gm::generate\_init\_visgen:

|  |  |
| --- | --- |
| Public Member Functions | |
|  | generate\_init\_visgen (std::ostream &o) |
|  | |
| void | operator() (nil const &) const |
|  | |
| void | operator() (int\_var\_decl const &x) const |
|  | |
| template<typename D > | |
| std::string | function\_args (const std::string &fun\_prefix, const D &x) const |
|  | |
| void | operator() (double\_var\_decl const &x) const |
|  | |
| void | operator() (vector\_var\_decl const &x) const |
|  | |
| void | operator() (row\_vector\_var\_decl const &x) const |
|  | |
| void | operator() (matrix\_var\_decl const &x) const |
|  | |
| void | operator() (unit\_vector\_var\_decl const &x) const |
|  | |
| void | operator() (simplex\_var\_decl const &x) const |
|  | |
| void | operator() (ordered\_var\_decl const &x) const |
|  | |
| void | operator() (positive\_ordered\_var\_decl const &x) const |
|  | |
| void | operator() (cholesky\_factor\_var\_decl const &x) const |
|  | |
| void | operator() (cholesky\_corr\_var\_decl const &x) const |
|  | |
| void | operator() (cov\_matrix\_var\_decl const &x) const |
|  | |
| void | operator() (corr\_matrix\_var\_decl const &x) const |
|  | |
| void | generate\_write\_loop (const std::string &write\_method\_name, const std::string &var\_name, const std::vector< expression > &dims) const |
|  | |
| void | generate\_name\_dims (const std::string name, size\_t num\_dims) const |
|  | |
| void | generate\_declaration (const std::string &name, const std::string &base\_type, const std::vector< expression > &dims, const expression &type\_arg1=expression(), const expression &type\_arg2=expression()) const |
|  | |
| void | generate\_indent\_num\_dims (size\_t base\_indent, const std::vector< expression > &dims, const expression &dim1, const expression &dim2) const |
|  | |
| void | generate\_buffer\_loop (const std::string &base\_type, const std::string &name, const std::vector< expression > &dims, const expression &dim1=expression(), const expression &dim2=expression(), int indent=2U) const |
|  | |
| void | generate\_dims\_loop\_fwd (const std::vector< expression > &dims, int indent=2U) const |
|  | |
| void | generate\_check\_int (const std::string &name, size\_t) const |
|  | |
| void | generate\_check\_double (const std::string &name, size\_t) const |
|  | |
| Public Member Functions inherited from stan::gm::visgen | |
|  | visgen (std::ostream &o) |
|  | |

|  |  |
| --- | --- |
| Public Attributes | |
| var\_size\_validating\_visgen | var\_size\_validator\_ |
|  | |
| Public Attributes inherited from stan::gm::visgen | |
| std::ostream & | o\_ |
|  | |

|  |  |
| --- | --- |
| Additional Inherited Members | |
| Public Types inherited from stan::gm::visgen | |
| typedef void | result\_type |
|  | |

## Detailed Description

Definition at line 2560 of file generator.hpp.

## Constructor & Destructor Documentation

|  |  |  |  |  |  |
| --- | --- | --- | --- | --- | --- |
| stan::gm::generate\_init\_visgen::generate\_init\_visgen | ( | std::ostream & | *o* | ) |  |

inline

Definition at line 2562 of file generator.hpp.

## Member Function Documentation

template<typename D >

|  |  |  |  |
| --- | --- | --- | --- |
| std::string stan::gm::generate\_init\_visgen::function\_args | ( | const std::string & | *fun\_prefix*, |
|  |  | const D & | *x* |
|  | ) |  | const |

inline

Definition at line 2575 of file generator.hpp.

|  |  |  |  |
| --- | --- | --- | --- |
| void stan::gm::generate\_init\_visgen::generate\_buffer\_loop | ( | const std::string & | *base\_type*, |
|  |  | const std::string & | *name*, |
|  |  | const std::vector< expression > & | *dims*, |
|  |  | const expression & | *dim1* = `expression()`, |
|  |  | const expression & | *dim2* = `expression()`, |
|  |  | int | *indent* = `2U` |
|  | ) |  | const |

inline

Definition at line 2722 of file generator.hpp.

|  |  |  |  |
| --- | --- | --- | --- |
| void stan::gm::generate\_init\_visgen::generate\_check\_double | ( | const std::string & | *name*, |
|  |  | size\_t |  |
|  | ) |  | const |

inline

Definition at line 2784 of file generator.hpp.

|  |  |  |  |
| --- | --- | --- | --- |
| void stan::gm::generate\_init\_visgen::generate\_check\_int | ( | const std::string & | *name*, |
|  |  | size\_t |  |
|  | ) |  | const |

inline

Definition at line 2776 of file generator.hpp.

|  |  |  |  |
| --- | --- | --- | --- |
| void stan::gm::generate\_init\_visgen::generate\_declaration | ( | const std::string & | *name*, |
|  |  | const std::string & | *base\_type*, |
|  |  | const std::vector< expression > & | *dims*, |
|  |  | const expression & | *type\_arg1* = `expression()`, |
|  |  | const expression & | *type\_arg2* = `expression()` |
|  | ) |  | const |

inline

Definition at line 2703 of file generator.hpp.

|  |  |  |  |
| --- | --- | --- | --- |
| void stan::gm::generate\_init\_visgen::generate\_dims\_loop\_fwd | ( | const std::vector< expression > & | *dims*, |
|  |  | int | *indent* = `2U` |
|  | ) |  | const |

inline

Definition at line 2765 of file generator.hpp.

|  |  |  |  |
| --- | --- | --- | --- |
| void stan::gm::generate\_init\_visgen::generate\_indent\_num\_dims | ( | size\_t | *base\_indent*, |
|  |  | const std::vector< expression > & | *dims*, |
|  |  | const expression & | *dim1*, |
|  |  | const expression & | *dim2* |
|  | ) |  | const |

inline

Definition at line 2714 of file generator.hpp.

|  |  |  |  |
| --- | --- | --- | --- |
| void stan::gm::generate\_init\_visgen::generate\_name\_dims | ( | const std::string | *name*, |
|  |  | size\_t | *num\_dims* |
|  | ) |  | const |

inline

Definition at line 2697 of file generator.hpp.

|  |  |  |  |
| --- | --- | --- | --- |
| void stan::gm::generate\_init\_visgen::generate\_write\_loop | ( | const std::string & | *write\_method\_name*, |
|  |  | const std::string & | *var\_name*, |
|  |  | const std::vector< expression > & | *dims* |
|  | ) |  | const |

inline

Definition at line 2687 of file generator.hpp.

|  |  |  |  |  |  |
| --- | --- | --- | --- | --- | --- |
| void stan::gm::generate\_init\_visgen::operator() | ( | nil const & |  | ) | const |

inline

Definition at line 2566 of file generator.hpp.

|  |  |  |  |  |  |
| --- | --- | --- | --- | --- | --- |
| void stan::gm::generate\_init\_visgen::operator() | ( | int\_var\_decl const & | *x* | ) | const |

inline

Definition at line 2567 of file generator.hpp.

|  |  |  |  |  |  |
| --- | --- | --- | --- | --- | --- |
| void stan::gm::generate\_init\_visgen::operator() | ( | double\_var\_decl const & | *x* | ) | const |

inline

Definition at line 2599 of file generator.hpp.

|  |  |  |  |  |  |
| --- | --- | --- | --- | --- | --- |
| void stan::gm::generate\_init\_visgen::operator() | ( | vector\_var\_decl const & | *x* | ) | const |

inline

Definition at line 2607 of file generator.hpp.

|  |  |  |  |  |  |
| --- | --- | --- | --- | --- | --- |
| void stan::gm::generate\_init\_visgen::operator() | ( | row\_vector\_var\_decl const & | *x* | ) | const |

inline

Definition at line 2615 of file generator.hpp.

|  |  |  |  |  |  |
| --- | --- | --- | --- | --- | --- |
| void stan::gm::generate\_init\_visgen::operator() | ( | matrix\_var\_decl const & | *x* | ) | const |

inline

Definition at line 2623 of file generator.hpp.

|  |  |  |  |  |  |
| --- | --- | --- | --- | --- | --- |
| void stan::gm::generate\_init\_visgen::operator() | ( | unit\_vector\_var\_decl const & | *x* | ) | const |

inline

Definition at line 2631 of file generator.hpp.

|  |  |  |  |  |  |
| --- | --- | --- | --- | --- | --- |
| void stan::gm::generate\_init\_visgen::operator() | ( | simplex\_var\_decl const & | *x* | ) | const |

inline

Definition at line 2638 of file generator.hpp.

|  |  |  |  |  |  |
| --- | --- | --- | --- | --- | --- |
| void stan::gm::generate\_init\_visgen::operator() | ( | ordered\_var\_decl const & | *x* | ) | const |

inline

Definition at line 2645 of file generator.hpp.

|  |  |  |  |  |  |
| --- | --- | --- | --- | --- | --- |
| void stan::gm::generate\_init\_visgen::operator() | ( | positive\_ordered\_var\_decl const & | *x* | ) | const |

inline

Definition at line 2652 of file generator.hpp.

|  |  |  |  |  |  |
| --- | --- | --- | --- | --- | --- |
| void stan::gm::generate\_init\_visgen::operator() | ( | cholesky\_factor\_var\_decl const & | *x* | ) | const |

inline

Definition at line 2659 of file generator.hpp.

|  |  |  |  |  |  |
| --- | --- | --- | --- | --- | --- |
| void stan::gm::generate\_init\_visgen::operator() | ( | cholesky\_corr\_var\_decl const & | *x* | ) | const |

inline

Definition at line 2666 of file generator.hpp.

|  |  |  |  |  |  |
| --- | --- | --- | --- | --- | --- |
| void stan::gm::generate\_init\_visgen::operator() | ( | cov\_matrix\_var\_decl const & | *x* | ) | const |

inline

Definition at line 2673 of file generator.hpp.

|  |  |  |  |  |  |
| --- | --- | --- | --- | --- | --- |
| void stan::gm::generate\_init\_visgen::operator() | ( | corr\_matrix\_var\_decl const & | *x* | ) | const |

inline

Definition at line 2680 of file generator.hpp.

## Member Data Documentation

var\_size\_validating\_visgen stan::gm::generate\_init\_visgen::var\_size\_validator\_

Definition at line 2561 of file generator.hpp.

---

The documentation for this struct was generated from the following file:

- src/stan/gm/generator.hpp


---

[ Stan Home Page ]

*© 2011–2014,
Stan Development Team.*
